# Supplementary material for: Dynalign II: common secondary structure prediction for RNA homologs with domain insertions
Source: Nucleic Acids Res. 2014 Nov 21;42(22):13939–48. doi: 10.1093/nar/gku1172 (PMC4267632; doi:10.1093/nar/gku1172)
Supplement: SUPPLEMENTARY DATA [file supp_gku1172_nar-02021-z-2014-File012.zip › manual/RNA_class/html/class_oligowalk__object.html]

RNAstructure Classes: Oligowalk\_object Class Reference

- Main Page
- Classes
- Files

- Class List
- Class Hierarchy
- Class Members

# Oligowalk\_object Class Reference

Oligowalk\_object Class.
More...

`#include <Oligowalk_object.h>`

Inherits RNA.

List of all members.

|  |  |
| --- | --- |
|  |  |
| Public Member Functions | |
|  | Oligowalk\_object (const char sequence[]) |
|  | Constructor - user provides a sequence as a c string. |
|  | Oligowalk\_object (const char filename[], const int type) |
|  | Constructor - user provides a filename for existing file as a c string. |
|  | Oligowalk\_object (const bool IsRNA=true) |
|  | Default Constructor - user provides nothing. |
| int | Oligowalk (const int oligo\_length, const bool isDNA, const int option, const double oligo\_concentration, const int usesub, const int start, const int stop) |
| double | GetBreakTargetDG (const int index) |
| double | GetDuplexDG (const int index) |
| double | GetOligoOligoDG (const int index) |
| double | GetOligoSelfDG (const int index) |
| double | GetOverallDG (const int index) |
| double | GetTm (const int index) |
| int | WriteReport (const char outputfilename[], const int oligo\_length, const bool isDNA, const int option, const double oligo\_concentration, const int usesub, const int start, const int stop) |
| int | OligoScreen (const char infilename[], const char outfilename[]) |
|  | This function runs OligoScreen. |
| char \* | GetErrorMessage (const int error) |
|  | ~Oligowalk\_object () |
|  | This is the destructor. |
| Private Member Functions | |
| void | CommonConstructor () |
| Private Attributes | |
| int \*\* | table |
| int \*\* | numofsubstructures |
| siPREFILTER \* | prefilter |
| int | length |

---

## Detailed Description

Oligowalk\_object Class.

The Oligowalk\_class inhereits from RNA and provides the OligoWalk functionality. Additionally, it provides OligoScreen. 

---

## Constructor & Destructor Documentation

|  |  |  |  |  |  |
| --- | --- | --- | --- | --- | --- |
| Oligowalk\_object::Oligowalk\_object | ( | const char | *sequence*[] | ) |  |

Constructor - user provides a sequence as a c string.

This constructor simply calls the underlying RNA(const char sequence[], const bool IsRNA=true) constructor and sets IsRNA=true. Input sequence should contain A,C,G,T,U,a,c,g,t,u,x,X. Capitalization makes no difference. T=t=u=U. U is assumed by OligoWalk. x=X= nucleotide that neither stacks nor pairs. For now, any unknown nuc is considered 'X'. Note that sequences will subsequently be indexed starting at 1 (like a biologist), so that the 0th position in the sequence array will be nucleotide 1.

**Parameters:**
:   |  |  |  |
    | --- | --- | --- |
    |  | *sequence* | is a NULL terminated c string. |

|  |  |  |  |  |
| --- | --- | --- | --- | --- |
| Oligowalk\_object::Oligowalk\_object | ( | const char | *filename*[], |  |
|  |  | const int | *type* |  |
|  | ) |  |  |  |

Constructor - user provides a filename for existing file as a c string.

This constructor simply calls the underlying RNA(const char filename[], const int type, const bool IsRNA=true) constructor and sets IsRNA=true. The existing file, specified by filename, can either be a ct file, a sequence, or an RNAstructure save file. Therefore, the user provides a flag for the file: type = 1 => .ct file, type = 2 => .seq file, type = 3 => partition function save (.pfs) file, type = 4 => folding save file (.sav). For OligoWalk calculations, types 1 and 2 are the relevant types. This constructor generates internal error codes that can be accessed by GetErrorCode() after the constructor is called. 0 = no error. The errorcode can be resolved to a c string using GetErrorMessage. Note also that save files explicitly store the thermodynamic parameters, therefore changing the backbone type as compaared to the original calculation will not change structure predictions.

**Parameters:**
:   |  |  |  |
    | --- | --- | --- |
    |  | *filename* | is null terminated c string. |
    |  | *type* | is an integer that indicates the file type. |

|  |  |  |  |  |  |
| --- | --- | --- | --- | --- | --- |
| Oligowalk\_object::Oligowalk\_object | ( | const bool | *IsRNA* = `true` | ) |  |

Default Constructor - user provides nothing.

This constructor calls the underlying RNA(IsRNA=true) constructor. This basic constructor is provided for performing OligoScreen calculations, which do not need an input sequence. This constructor needs to be called with RNA=true for Oligos to be RNA and RNA=false for Oligos to be DNA.

**Parameters:**
:   |  |  |  |
    | --- | --- | --- |
    |  | *IsRNA* | is a bool where true=RNA and false=DNA. |

|  |  |  |  |  |
| --- | --- | --- | --- | --- |
| Oligowalk\_object::~Oligowalk\_object | ( |  | ) |  |

This is the destructor.

---

## Member Function Documentation

|  |  |  |  |  |
| --- | --- | --- | --- | --- |
| void Oligowalk\_object::CommonConstructor | ( |  | ) | `[private]` |

|  |  |  |  |  |  |
| --- | --- | --- | --- | --- | --- |
| double Oligowalk\_object::GetBreakTargetDG | ( | const int | *index* | ) |  |

Get the breaking target DG for a given nucleotide. This can only be called after performing a valid OligoWalk calculation. In case of error, the function returns a free energy change of zero. Note!: That a free energy change of zero is also a valid folding free energy change. Errors will also generate an internal error code, accessible with GetErrorCode().

**Parameters:**
:   |  |  |  |
    | --- | --- | --- |
    |  | *index* | is an int that specifies the 5' end of an oligonucleotide binding site on the target. |

**Returns:**
:   A double that is the free energy change in kcal/mol.

|  |  |  |  |  |  |
| --- | --- | --- | --- | --- | --- |
| double Oligowalk\_object::GetDuplexDG | ( | const int | *index* | ) |  |

Get the duplex DG for a given nucleotide. This can only be called after performing a valid OligoWalk calculation. In case of error, the function returns a free energy change of zero. Note!: That a free energy change of zero is also a valid folding free energy change. Errors will also generate an internal error code, accessible with GetErrorCode().

**Parameters:**
:   |  |  |  |
    | --- | --- | --- |
    |  | *index* | is an int that specifies the 5' end of an oligonucleotide binding site on the target. |

**Returns:**
:   A double that is the free energy change in kcal/mol.

|  |  |  |  |  |  |
| --- | --- | --- | --- | --- | --- |
| char \* Oligowalk\_object::GetErrorMessage | ( | const int | *error* | ) |  |

Return error messages based on code from GetErrorCode and other error codes. 100 = no OligoWalk data present 101 = OligoWalk has already been performed other codes are handled by the RNA base class function GetErrorMessage.

**Parameters:**
:   |  |  |  |
    | --- | --- | --- |
    |  | *error* | is the integer error code provided by GetErrorCode()/ |

**Returns:**
:   A pointer to a c string that provides an error message or from other functions that return integer error codes.

Reimplemented from RNA.

|  |  |  |  |  |  |
| --- | --- | --- | --- | --- | --- |
| double Oligowalk\_object::GetOligoOligoDG | ( | const int | *index* | ) |  |

Get the bimolecular oligo-oligo DG for a given nucleotide. This can only be called after performing a valid OligoWalk calculation. In case of error, the function returns a free energy change of zero. Note!: That a free energy change of zero is also a valid folding free energy change. Errors will also generate an internal error code, accessible with GetErrorCode().

**Parameters:**
:   |  |  |  |
    | --- | --- | --- |
    |  | *index* | is an int that specifies the 5' end of an oligonucleotide binding site on the target. |

**Returns:**
:   A double that is the free energy change in kcal/mol.

|  |  |  |  |  |  |
| --- | --- | --- | --- | --- | --- |
| double Oligowalk\_object::GetOligoSelfDG | ( | const int | *index* | ) |  |

Get the oligo-self DG for a given nucleotide. This can only be called after performing a valid OligoWalk calculation. In case of error, the function returns a free energy change of zero. Note!: That a free energy change of zero is also a valid folding free energy change. Errors will also generate an internal error code, accessible with GetErrorCode().

**Parameters:**
:   |  |  |  |
    | --- | --- | --- |
    |  | *index* | is an int that specifies the 5' end of an oligonucleotide binding site on the target. |

**Returns:**
:   A double that is the free energy change in kcal/mol.

|  |  |  |  |  |  |
| --- | --- | --- | --- | --- | --- |
| double Oligowalk\_object::GetOverallDG | ( | const int | *index* | ) |  |

Get the overall DG for a given nucleotide. This can only be called after performing a valid OligoWalk calculation. In case of error, the function returns a free energy change of zero. Note!: That a free energy change of zero is also a valid folding free energy change. Errors will also generate an internal error code, accessible with GetErrorCode().

**Parameters:**
:   |  |  |  |
    | --- | --- | --- |
    |  | *index* | is an int that specifies the 5' end of an oligonucleotide binding site on the target. |

**Returns:**
:   A double that is the free energy change in kcal/mol.

|  |  |  |  |  |  |
| --- | --- | --- | --- | --- | --- |
| double Oligowalk\_object::GetTm | ( | const int | *index* | ) |  |

Get the Tm for a given nucleotide. This can only be called after performing a valid OligoWalk calculation. In case of error, the function returns a free energy change of zero. Note!: That a free energy change of zero is also a valid folding free energy change. Errors will also generate an internal error code, accessible with GetErrorCode().

**Parameters:**
:   |  |  |  |
    | --- | --- | --- |
    |  | *index* | is an int that specifies the 5' end of an oligonucleotide binding site on the target. |

**Returns:**
:   A double that is the Tm in degrees C.

|  |  |  |  |  |
| --- | --- | --- | --- | --- |
| int Oligowalk\_object::OligoScreen | ( | const char | *infilename*[], |  |
|  |  | const char | *outfilename*[] |  |
|  | ) |  |  |  |

This function runs OligoScreen.

Read a list of oligonucleotides in infilename and output thermodynamic characteristics in outfilename. The backbone type is set when the constructor is called. Note that oligoscreen has no target sequence, so the default constructor OligoWalk(bool IsRNA) should be used.

**Parameters:**
:   |  |  |  |
    | --- | --- | --- |
    |  | *infilename* | is a c-string that indicates the filename to be read. |
    |  | *outfilename* | is a c-string that indicates the name of an output file to be written with report. |

**Returns:**
:   An int that indicates an error code that can be parsed by GetErrorMessage() or GetErrorMessageString(), 0 = no error.

|  |  |  |  |  |
| --- | --- | --- | --- | --- |
| int Oligowalk\_object::Oligowalk | ( | const int | *oligo\_length*, |  |
|  |  | const bool | *isDNA*, |  |
|  |  | const int | *option*, |  |
|  |  | const double | *oligo\_concentration*, |  |
|  |  | const int | *usesub*, |  |
|  |  | const int | *start*, |  |
|  |  | const int | *stop* |  |
|  | ) |  |  |  |

Perform an OligoWalk calculation. Note that this can only be performed once.

**Parameters:**
:   |  |  |  |
    | --- | --- | --- |
    |  | *oligo\_length* | is an int that gives the length of the oligonucleotides. |
    |  | *isDNA* | is a bool that indicates the oligonucleotide chemistry, where true = DNA and false = RNA. |
    |  | *option* | is an int that gives the calculation type, where option 1 = break local target structure to bind oligo, option 2 = refold target RNA after oligo binding, and option 3 = no target structure considered. |
    |  | *oligo\_concentration* | is a double that indicates the oligonucleotide concentration in M. |
    |  | *usesub* | is an int that indicates whether suboptimal structures are to be used, where 0 = none and 3 = use heuristic method. |
    |  | *start* | is an int that indicates the starting location of the walk. |
    |  | *stop* | is an int that indicates the ending location of the walk. |

**Returns:**
:   An integer that indicates an error code that can be parsed by GetErrorMessage() or GetErrorMessageString(), 0 = no error.

|  |  |  |  |  |
| --- | --- | --- | --- | --- |
| int Oligowalk\_object::WriteReport | ( | const char | *outputfilename*[], |  |
|  |  | const int | *oligo\_length*, |  |
|  |  | const bool | *isDNA*, |  |
|  |  | const int | *option*, |  |
|  |  | const double | *oligo\_concentration*, |  |
|  |  | const int | *usesub*, |  |
|  |  | const int | *start*, |  |
|  |  | const int | *stop* |  |
|  | ) |  |  |  |

Write a report for an OligoWalk calculation. This must be called after performing a valid OligoWalk calculation.

**Parameters:**
:   |  |  |  |
    | --- | --- | --- |
    |  | *outputfilename* | is a c-string that provides a filename to which the report will be written. |
    |  | *oligo\_length* | is an int that gives the length of the oligonucleotides. |
    |  | *isDNA* | is a bool that indicates the oligonucleotide chemistry, where true = DNA and false = RNA. |
    |  | *option* | is an int that gives the calculation type, where option 1 = break local target structure to bind oligo, option 2 = refold target RNA after oligo binding, and option 3 = no target structure considered. |
    |  | *oligo\_concentration* | is a double that indicates the oligonucleotide concentration in M. |
    |  | *usesub* | is an int that indicates whether suboptimal structures are to be used, where 0 = none and 3 = use heuristic method. |
    |  | *start* | is an int that indicates the starting location of the walk. |
    |  | *stop* | is an int that indicates the ending location of the walk. |

**Returns:**
:   An integer that indicates an error code that can be parsed by GetErrorMessage() or GetErrorMessageString(), 0 = no error.

---

## Member Data Documentation

int Oligowalk\_object::length `[private]`

int \*\* Oligowalk\_object::numofsubstructures `[private]`

siPREFILTER\* Oligowalk\_object::prefilter `[private]`

int\*\* Oligowalk\_object::table `[private]`

---

The documentation for this class was generated from the following files:

- F:/RNAstructure-co-from-repository/RNAstructure/RNA\_class/Oligowalk\_object.h- F:/RNAstructure-co-from-repository/RNAstructure/RNA\_class/Oligowalk\_object.cpp

---

Generated on Tue Jul 30 10:52:29 2013 for RNAstructure Classes by 
 1.5.7.1
